# Detecting suicidal risk using MMPI-2 based on machine learning algorithm

**DOI:** 10.1038/s41598-021-94839-5

**Published:** 2021-07-28

**Authors:** Sunhae Kim, Hye-Kyung Lee, Kounseok Lee

**Affiliations:** 1 Department of Psychiatry, Hanyang University Medical Center, 222-1, Wangsimni-ro, Seongdong-gu, Seoul, 04763 Republic of Korea; 2 Department of Nursing, College of Nursing and Health, Kongju National University, Gongju, Republic of Korea

**Keywords:** Human behaviour, Psychology, Risk factors, Mathematics and computing, Psychiatric disorders

## Abstract

Minnesota Multiphasic Personality Inventory-2 (MMPI-2) is a widely used tool for early detection of psychological maladjustment and assessing the level of adaptation for a large group in clinical settings, schools, and corporations. This study aims to evaluate the utility of MMPI-2 in assessing suicidal risk using the results of MMPI-2 and suicidal risk evaluation. A total of 7,824 datasets collected from college students were analyzed. The MMPI-2-Resturcutred Clinical Scales (MMPI-2-RF) and the response results for each question of the Mini International Neuropsychiatric Interview (MINI) suicidality module were used. For statistical analysis, random forest and K-Nearest Neighbors (KNN) techniques were used with suicidal ideation and suicide attempt as dependent variables and 50 MMPI-2 scale scores as predictors. On applying the random forest method to suicidal ideation and suicidal attempts, the accuracy was 92.9% and 95%, respectively, and the Area Under the Curves (AUCs) were 0.844 and 0.851, respectively. When the KNN method was applied, the accuracy was 91.6% and 94.7%, respectively, and the AUCs were 0.722 and 0.639, respectively. The study confirmed that machine learning using MMPI-2 for a large group provides reliable accuracy in classifying and predicting the subject's suicidal ideation and past suicidal attempts.

## Introduction

Machine learning (ML) is defined as a computational strategy that automatically determines methods and parameters to arrive at an optimal solution to a problem, rather than preprogramming by humans to present a fixed solution^1^. Moreover, machine learning algorithms are integrated into everyday life as internet searches and product recommendations, translation services, speech recognition services, and autonomous vehicles^2^.

Machine learning is the study and application of algorithms and systems that can improve knowledge or performance through experience. The basic premise of machine learning is the assumption that a machine can learn from data, recognize patterns in data, and understand data with minimal human intervention. By making the data understandable from the start, machines can detect complex and meaningful data patterns, which may be difficult or impossible for humans to derive. Furthermore, machine learning algorithms can be changed and improved when exposed to new data, so these detection patterns have the advantages of efficiency, complexity, and flexibility^3^.

In psychiatry, machine learning applications have been proposed to improve diagnostic and prognostic accuracy and determine treatment options^4^. In recent studies, machine learning is applied to big data in medical and health fields for disease diagnosis, treatment, and prevention^5^. Machine learning is specifically useful in predicting human behavior, including high-risk behavior. It can be applied to improve the effectiveness and goals of prevention programs and interventions^1^.

Some studies apply machine learning to differentiate between various types of psychopathology. For example, some studies use machine learning, the Structured Inventory of Malingered Symptomatology (SIMS) scale^6^, and the Minnesota Multiphasic Personality Inventory-2 (MMPI-2) scale to discriminate malingering to obtain external benefits^7^. Several studies have been conducted on MMPI-2 in particular as it is useful and expandable^8^. Regarding prediction, machine learning technology has advantages in accuracy and scalability compared to conventional statistical approaches^3^. Hence, various machine learning studies are focused on suicide prediction^3,5,9–12^.

Suicide results from a combination of factors derived from genetic, neurobiological, psychological, and social factors^13–15^. Suicidal behavior, attempt and completion are closely related to impulsivity and aggression^16^. And they share many neurobiological correlates^14,17–19^, comorbidity of psychiatric disorders such as mood disorder, borderline personality disorder, and substance use disorder (SUD)^20–28^. Therefore, it will be important to screen various risk factors and psychopathology to determine suicide risk.

Methods of screening for suicide may include unsystematic interviews, systematically structured or semi-structured interviews, and the use of self-report tests. The screening scale used by a clinician may be appropriate in a clinical environment, such as inpatient or outpatient situations. However, there are many limitations to the amount of time spent on screening tests for a large number of groups. Moreover, the self-report test is suitable for screening as it can be performed with ease, but the test validity may be a problem depending on the examinee’s attitude.

Among the self-report tests, MMPI-2 is one of the most widely used objective personality tests worldwide and is the most frequently used scale for evaluating psychopathology and emotional function^29,30^. MMPI-2 is very useful in distinguishing psychiatric disorders. It is frequently used for assessing clinical conditions related to suicidal risk^31^. Specifically, it has a validity scale to detect inappropriate examinee attitudes and judge the interpretability test data.

Many studies have been conducted to screen suicidal risk using MMPI-2. Studies report that some clinical scales are associated with suicidal ideation and behaviors, but elevated clinical scale scores show inconsistent results^32–36^.

The authors examined the difference in suicidal risk using the MMPI-2 reconstructed clinical scale, beyond the inconsistent results in previous studies on suicidal risk and clinical scales^37^. Compared with the control group, all of the suicidal risk group showed an overall increase in the Minnesota Multiphasic Personality Inventory-2-Restructured Clinical (MMPI-2-RC) scale, which confirmed that various psychopathological characteristics were overlapped with suicidal risk. However, this rise only confirms the tendency, and there is still insufficient evidence on the predictability of suicide-related pathology.

The MMPI-2, which is widely used in medical fields (psychiatric treatment sites and health check-ups) and employee selection, can assist in suicide prevention by classifying and predicting high suicidal risk. Therefore, this study aimed to distinguish people with suicidal risk by applying the latest machine learning algorithms using MMPI-2 results.

## Results

**Figure 1:**
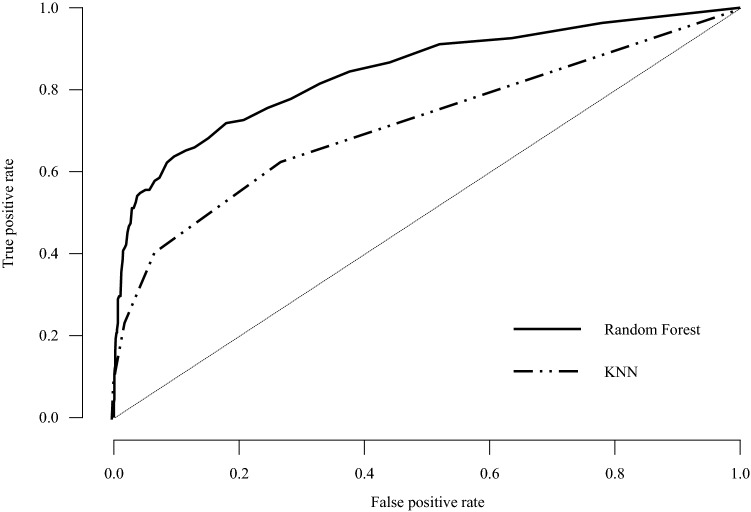
ROC curve plots for suicidal ideation.

**Figure 2:**
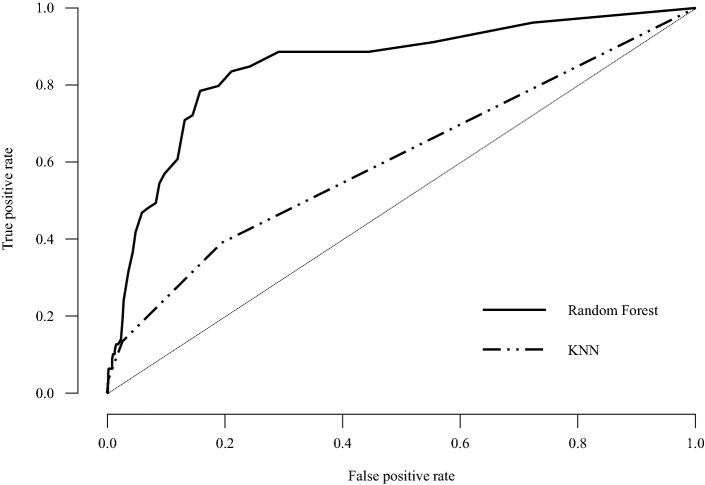
ROC curve plots for suicidal attempt.

Among the 7824 participants, 3685 (47.1%) were male, a total of 673 (8.6%) participants classified as a suicidal ideation group, and 404 (5.4%) were classified as a suicidal attempt group (Table 1). Of the total datasets, 5008 were used as train data, 1252 as validation data, and 1564 as test data. Prediction accuracy of the random forest method was 92.9% for suicidal ideation and 95% for suicidal attempts; k-Nearest Neighbors (KNN) accurately predicted 91.6% of suicidal ideation and 94.7% of suicidal attempts (Table 2). Table 3 shows all parameters for suicidal ideation and suicidal attempts. When using the Suicidal/Death Ideation (SUI) scale t score to predict suicidal ideation and suicidal attempts, the area under the curve (AUCs) were 0.769 and 0.815. And using the random forest method to predict suicidal ideation and suicidal attempts, the AUCs were 0.844 and 0.851, which were more accurate than 0.722 and 0.639 when KNN was applied (Table 3, Figs. 1, 2). The F1 score was highest when using the random forest method of suicide attempt (92.6%) and lowest when applying KNN for suicide ideation (88.4%, Table 3).

**Table 1:** General characteristics of the participants (n = 7824).

Factor	Value
Sex (male)	3685 (47.1%)
Age	19.57 ± 1.27
Suicidal ideation	673 (8.6%)
Previous attempt	404 (5.2%)

Values were presented as mean ± SD or n (%).

**Table 2:** Accuracy by ML methods.

	ML method	Trees or nearest neighbors	Predictors per split	Validation accuracy	Test accuracy	OOB accuracy
Suicidal ideation	Random forest	72	7	0.920	0.929	0.206
	KNN	9		0.922	0.916	
Suicidal attempt	Random forest	90	7	0.940	0.950	0.016
	KNN	9		0.943	0.947	

The Random Forest models are optimized with respect to the out-of-bag accuracy. The kNN models are optimized with respect to the validation set accuracy.

**Table 3:** Evaluation metrics.

Outcome variable	ML methods	Precision	Recall	F1 score	AUC
Suicidal ideation	Random forest	0.920	0.929	0.912	0.844
	KNN	0.913	0.916	0.884	0.722
Suicidal attempt	Random forest	0.953	0.950	0.926	0.851
	KNN	0.897	0.947	0.921	0.639

Area under curve (AUC) is calculated for every class against all other classes.

## Discussion

This study attempted to predict and report suicide-related risk with the ML technique using 50 scales of MMPI-2, the most commonly used self-report evaluation tool. Although differences exist depending on the ML technique used, it was confirmed that each predicted suicidal ideation and past suicidal attempts at an excellent level. In particular, in the case of the random forest method, AUC of 0.844 for suicidal ideation and 0.851 for suicidal attempts represent good performance values, indicating the potential for prediction using machine learning techniques without directly checking suicidal ideation and suicidal attempts. Research studies that predicted suicide accidents using public health data along with recent machine learning algorithms (AUC = 0.85)^5^ or predicted suicide accidents using various self-reporting tools and socio-demographic statistics data (AUC = 0.87–0.91)^10^, and AUC in this study show similar performance.

Machine learning technology for suicide prediction has an edge in accuracy and scalability compared to conventional statistical approaches^3^. Despite these advantages, there is a limitation that it has not yet been able to produce accurate predictions repeatedly due to the potential complexity of suicidal ideation and actions^3,12^. In a recent study investigating the probability of death due to suicide using insurance data and general characteristics of the National Health Insurance Service cohort in Korea, the machine learning model predicting death due to suicide showed a low-performance value (AUC = 0.68)^38^.

Previous suicidal attempts are the strongest predictor of future suicidal attempts^12,39^, but the AUC values vary depending on the time and measurement of past suicidal attempts (AUC 65–91%)^10,11^, and so previous study decided to conduct a machine learning study by setting suicidal ideation as a better potential predictor of suicidal risk than suicidal attempts^5^. Therefore, this paper has the advantage of applying machine learning predictions by setting both suicidal attempts and suicidal ideation as parameters, which are potential predictors of suicidal risk, and verified the prediction of machine learning by comparing various techniques.

Currently, machine learning risk algorithms can predict who will attempt or die by suicide but cannot tell when a person at risk can act. If the risk of suicide is considered high enough to threaten the individual's safety, clinicians must take steps to intervene, which in many cases may include involuntary hospitalization. This decision is one of the most difficult predictions, and clinicians are responsible for determining the risk level, given the limitations of existing algorithms^12^. Therefore, more information and knowledge will be required from the clinician about the influence level of various variables on suicidal risk, the timing of risk level, and intervention. For example, indirect tools such as ERQ, ARS, and SWLS represent better predictions of actual suicidal attempts than direct measures of suicidal ideation^10^. In many suicide accidents and suicidal attempts, the patients experience mood disorders or anxiety disorders^40–43^. The stress associated with academics, job, and life events is also related to suicide^44,45^. Traditional approaches to preventing and assessing suicide are generally expensive and time-consuming. As individuals at high suicidal risk often refuse to seek experts^46,47^, machine learning algorithms to predict suicide risk can be an effective alternative.

Accurate risk detection is necessary for suicide prevention, but studies to date have not yet verified the suitability of various risk management strategies in consideration of the suicidal risk level presented by the algorithm. Further, the most effective intervention for suicidal risk levels should be considered. However, no study has investigated the effect of intervention at the suicidal risk level suggested by the algorithm^3^. Further research is necessary for suicidal risk assessment and intervention by clinicians.

The random forest technique, which showed an excellent level of accuracy in this study, belongs to the unsupervised learning algorithm and has the advantage of being relatively easy to use because it only needs to determine the number of trees and the number of conditions that enter the branch points when creating a model^48,49^. However, a limitation is that one cannot obtain information other than the prediction result because the inside of the generated decision tree cannot be observed^48,50^. Moreover, machine learning cannot accurately describe the relationship between input and output^51^. Therefore, it is difficult to determine the complex effect of the selected characteristics on determining classification.

The limitations of this study are as follows. First, these results are not representative of the entire population, as the survey was conducted at one university. Second, as a self-reported study, there is a limit to fully trusting subject responses. Self-report tests are more open to suicide-related content than to standardized interviews. However, it seems necessary to analyze suicidal tendencies and psychopathological factors through various tools. Third, this study was conducted for a non-clinical group, and there was no clinical diagnosis and no information on the subject's psychiatric treatment history. This study was a retrospective analysis using data from part of a school project, and hence, it was difficult to obtain information. Fourth, there was no detailed suicide information on the fatality, method, and frequency of suicide. Fifth, because it is a cross-sectional study, the causal relationship between related factors and suicidal risk could not be clearly defined. In the future, it will be necessary to confirm through follow-up studies that continuously evaluate suicidal risk in various population groups, including clinical patients.

Nevertheless, this study was conducted on a large-scale, with consistent evaluation and multi-faceted analysis on the same group of college students, which may be its strongest point. There are many studies using MMPI-2, but this study verified its accuracy via additional evaluations related to suicide in a large group and confirmed the prediction potential with the subsequent use of MMPI-2 alone. In particular, it is possible to present the possibility of indirectly predicting and assessing the risk in a situation where it is difficult to directly ask questions on sensitive issues when evaluating the selection process of companies or schools or military enlistment. Moreover, as a study conducted at a single university, it is possible to identify risk factors through a long-term cohort group analysis through additional research projects.

The assessment of various types of psychopathology affecting suicide cannot be replaced by MMPI-2 alone. However, using MMPI-2, it is possible to obtain test results with secured validity for various aspects of psychopathology, and if used well together with clinical interviews, it may serve as an auxiliary tool. Furthermore, through the clinical characteristics of MMPI-2, this study uncovered various variables related to suicidal risk and various psychopathological factors influencing suicidal ideation and suicide accidents. If further analyzed, the possibility of using MMPI-2 in suicidal risk assessment is expected to increase.

## Conclusion

This study confirmed that ML using MMPI-2 provides reliable accuracy in classifying and predicting the subject's suicidal ideation and past suicidal attempts. Based on these findings, we believe that it will help clinicians detect and treat high-risk suicide groups early in practice.

## Methods

### Participants

**Figure 3:**
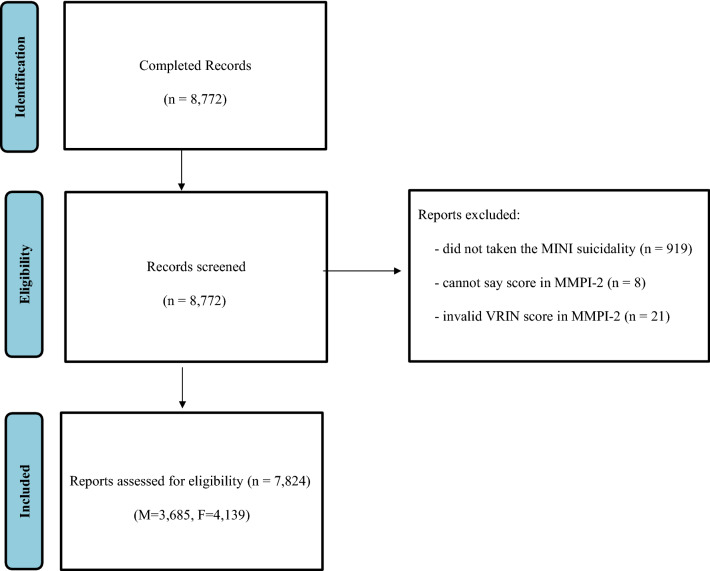
Flow chart of participants inclusion and exclusion.

This study used part of the questionnaire dataset from a student health check-up conducted at Kongju National University^37^. Written consent was obtained after explaining the purpose of the research to all subjects. The study analyzed the answers given by 7824 (3685 males, 4139 females) participants out of a total of 8772, excluding 948 participants (919 participants that did not take the MINI suicidality, 8 participants with 10 or more cannot say scores in MMPI-2, 21 participants have invalid VRIN score, Fig. 3). This study was approved by the National Kongju University Ethics Committee. The participants were informed that the information they provide would be kept strictly confidential and used for research purposes only, and written consent was obtained. This research involving human research participants must have been performed in accordance with the Declaration of Helsinki.

## Measurements

### Minnesota multiphasic personality inventory-2-restructured form

For Minnesota Multiphasic Personality Inventory-2 Restructured Form (MMPI-2-RF), a total of 50 scales that could effectively measure the clinical significance of MMPI-2 questions were developed and consisted of 8 validity scales and 42 major scales (Table 4). In this study, the Korean version of MMPI-2-RF was used, whose reliability and validity were verified^52^.

**Table 4:** The MMPI-2-RF variables used for machine learning.

Category	Abbreviation	Description
Validity indicators	VRIN-r	Variable response inconsistency
	TRIN-r	True response inconsistency
	F-r	Infrequent responses
	Fp-r	Infrequent psychopathology responses
	Fs	Infrequent somatic responses
	FBS-r	Symptom validity
	RBS	Response bias
	L-r	Uncommon virtues
	K-r	Adjustment validity
Higher-order (H-O) scales	EID	Emotional/internalizing dysfunction
	THD	Thought dysfunction
	BXD	Behavioral/externalizing dysfunction
Restructured clinical (RC) scales	RCd-(dem)	Demoralization
	RC1-(som)	Somatic complaints
	RC2-(lpe)	Low positive emotions
	RC3-(cyn)	Cynicism
	RC4-(asb)	Antisocial behavior
	RC6-(per)	Ideas of persecution
	RC7-(dne)	Dysfunctional negative emotions
	RC8-(abx)	Aberrant experiences
	RC9-(hpm)	Hypomanic activation
Somatic/cognitive scales	MLS	Malaise
	GIC	Gastro-intestinal complaints
	HPC	Head pain complaints
	NUC	Neurological complaints
	COG	Cognitive complaints
Internalizing scales	SUI	Suicidal/death ideation
	HLP	Helplessness/hopelessness
	SFD	Self-doubt
	NFC	Inefficacy
	STW	Stress/worry
	AXY	Anxiety
	ANP	Anger proneness
	BRF	Behavior-restricting fears
	MSF	Multiple specific fears
Externalizing scales	JCP	Juvenile conduct problems
	SUB	Substance abuse
	AGG	Aggression
	ACT	Activation
Interpersonal scales	FML	Family problems
	IPP	Interpersonal passivity
	SAV	Social avoidance
	SHY	Shyness
	DSF	Disaffiliativeness
Interest scales	AES	Aesthetic-literary interests
	MEC	Mechanical-physical interests
PSY-5 (personality psychopathology five) scales, revised	AGGR-r	Aggressiveness-revised
	PSYC-r	Psychoticism-revised
	DISC-r	Disconstraint-revised
	NEGE-r	Negative emotionality/neuroticism—revised
	INTR-r	Introversion/low positive emotionality-revised

### Suicide risk assessment

To evaluate suicidal risk, we used the MINI (Mini International Neuropsychiatric Interview, MINI) suicidality module. MINI is a structured interview tool developed in 1998 for diagnosing Diagnostic and Statistical Manual of Mental Disorders, fourth edition (DSM-IV) and the 10th revision of the International Statistical Classification of Diseases (ICD-10) Axis I mental illness. This study used the standardized version of Korean version 5.0^53^. Among these, suicide evaluation consisted of a total of 6 questions related to suicide, with weights for each question and the total score distributed from 0 to 29; the higher the score, the higher the suicidal risk. In this study, a subject was assigned to the suicide thought group on answering any one of the questions 1 to 3 related to suicidal ideation, and categorized in the suicide attempt group if the answer was yes to the sixth question on the case of a lifelong suicide attempt.

### Statistical analysis

To this end, MMPI-2-RF and suicide thought-related scales were used as inputs into the artificial neural network algorithm for student mental health check-up data to determine the factors affecting actual suicidal ideation. Among the machine learning techniques, Random forest classification and the KNN method were used.

There are two major importance indicators to measure the importance of explanatory variables in the random forest^54^. First, the Mean Decrease Gini (MDG) value is used as the average value from all trees by measuring the amount of impurity reduction of the selected variables each time each tree forming a random forest extends its branch. Therefore, a high MDG value for a specific variable means that classifying individuals with that variable helps to reduce impurity, that is, to group the same categories. Moreover, the importance of variables can be determined by the concept of accuracy, which is defined as Mean Decrease Accuracy (MDA). MDA is the average of the difference by variable between the accuracy of the constructed tree and the accuracy that decreases when reconstructed after removing a specific variable. The higher the influence of a variable in improving the classification accuracy, the greater is the amount of reduction in the accuracy on removing the variable. Thus, as the values of both indicators measuring the importance of variables in the random forest increase, the variable importance increases. The KNN algorithm has the same properties as the training data but extracts k data located closest to the training data using Euclidean distance from unclassified data and specifies the category of unclassified data through the class of the extracted data^55^.

The result variables were analyzed by suicidal ideation and suicidal attempts, using 50 scales of MMPI-2-RF as explanatory variables (Table 4). The AUC of receiver operating characteristic (ROC) curve was measured. The closer the AUC is to 1, the better is the model. The AUC 0.5 ~ 0.6 was evaluated as a coincidence level; 0.6 ~ 0.7 was not good, 0.7 ~ 0.8 was worthless, 0.8 ~ 0.9 was good, and 0.9 ~ 1.0 was excellent^56^.

A total 20% of the sample was used as test data, and 20% of the remaining 80% as validation data; each training data set and testing data set were randomly separated. All statistical analyses were performed using JASP v0.14.4 (Amsterdam, Netherland).
